# Menstrual Changes in Women Who Undergo Sleeve Gastrectomy in Saudi Arabia

**DOI:** 10.7759/cureus.66109

**Published:** 2024-08-04

**Authors:** Saeed Alsareii, Metrek Ali Almetrek, Saleh Hussain Alshaiban, Reem S Alshahrani, Najla A Alshahrani, Thikra E Atafi, Rasan F Almnjwami, Imtenan A Oberi, Reem H Al-Ruwaili

**Affiliations:** 1 Department of Surgery, College of Medicine, Najran University, Najran, SAU; 2 Department of Family Medicine, College of Medicine, Najran University, Najran, SAU; 3 College of Medicine, Najran University, Najran, SAU; 4 Department of Surgery, King Khalid University, Abha, SAU; 5 Faculty of Medicine, Jazan University, Jazan, SAU; 6 Department of General Surgery, King Faisal Medical Complex, Taif, SAU; 7 Department of General Surgery, Vision College, Riyadh, SAU

**Keywords:** post-operative changes, quality of life, women, saudi arabia, reproductive health, hormonal dynamics, laparoscopic sleeve gastrectomy, bariatric surgery, menstrual irregularities, obesity

## Abstract

Introduction

Obesity affects over 650 million globally, with rising rates posing significant public health challenges, especially among Saudi Arabian women. Obesity correlates with menstrual irregularities and reproductive health issues such as polycystic ovary syndrome (PCOS). Bariatric surgery (BS), particularly laparoscopic sleeve gastrectomy (LSG), is increasingly used due to its safety and effectiveness in treating obesity-related conditions. This study explores LSG's impact on menstrual cycles and fertility in Saudi women, aiming to optimize patient care and understand surgical effects on hormonal dynamics and reproductive health.

Methodology

It is a cross-sectional design among Saudi women post-sleeve gastrectomy from December 2023 to May 2024. Variables included age, marital status, and region, with primary outcomes focusing on menstrual cycle changes post surgery.

Results

Our study includes 387 participants, and demographic characteristics showed a significant proportion aged 26-35 years (n=147, 38.0%) and 36-45 years (n=119, 30.7%), with the majority being married (n=230, 59.4%). Regional distribution highlighted the south as the most represented (n=139, 35.9%), followed by the central (n=74, 19.1%). About 30.2% (n=117) reported chronic conditions. Post surgery, 70.5% (n=273) experienced menstrual changes, with regular cycles being the most common (n=102, 26.3%). Logistic regression indicated younger age as a protective factor against menstrual changes (p=0.028), while pre-surgery menstrual irregularities significantly predicted post-surgery changes (p=0.002). Regional analysis showed no significant association between geographic location and post-surgery menstrual changes (p=0.140). Overall, quality of life post-surgery was rated highly by participants, with 70.8% (n=274) giving ratings of 4 or 5.

Conclusion

Our study highlights a high prevalence of post-sleeve gastrectomy menstrual changes, predominantly regular cycles. Younger age appears protective, while pre-existing menstrual irregularities strongly predict postoperative changes. Regional differences did not significantly influence outcomes. Overall, participants reported high satisfaction with their quality of life post surgery.

## Introduction

Over 650 million people worldwide suffer from obesity, which not only impairs quality of life but also leads to several health problems [1]. Obesity has nearly tripled in prevalence in the past 50 years, endangering public health [2]. Obesity rates have increased across all age categories, with women and the elderly seeing the highest rates [3,4]. The exact cause of obesity is still unknown, but it is thought to be a mix of genetic, environmental, and physiological factors that affect things such as appetite, physical activity, and how the body handles energy balance [4,5]. The primary approach to addressing obesity is still to modify one's lifestyle while keeping body mass index (BMI) thresholds in mind. When a person's BMI is 30 or above, pharmaceutical therapies may aid in weight loss. However, for those with a BMI of BMI of 40 or more, or more than 35 who also have other medical issues, bariatric surgery (BS) is the advised course of action [4,6,7].

With more than 30% of adults (male and female) in Saudi Arabia reported to be obese and with 32% of women in Saudi Arabia considered overweight, the country is currently experiencing a serious obesity issue [8,9]. The high proportion of obesity among women in the Kingdom of Saudi Arabia is a significant problem as it impairs their ability to have offspring [8,10].

Because of their higher levels of estrogen, obese women are more likely to experience abnormalities and irregularities during their menstrual cycles [8,9]. Obese women frequently experience menstrual disorders, such as irregular periods and dysmenorrhea [11]. These anomalies could lead to conception problems [12]. Furthermore, it has been determined that obesity has a broad impact on the endocrine system and hormone regulation. Hormones indirectly regulate the development of endometriosis and the cycles associated with puberty and menstruation in a balanced way. This system becomes unbalanced due to obesity because adipose tissue interferes with the release of sex hormones. Additionally, obesity indirectly affects adipokines, insulin, and leptin levels [13].

Moreover, it has been reported that hormone imbalances linked to obesity worsen irregular menstruation and have a deleterious effect on reproductive health, including polycystic ovarian syndrome (PCOS) [14]. BS is a surgical weight loss alternative that is becoming increasingly popular as a conclusive and effective means of treating obesity [11]. Bariatric surgeries include operations such as sleeve gastrectomy (SG), adjustable gastric banding (LAGB), and Roux-en-Y gastric bypass (RYGB), which have proven to be an effective obesity treatment [15]. The most common surgery currently practiced is the laparoscopic SG (LSG). This treatment is chosen because of its well-established safety record, effectiveness, and noticeably shorter post-operative recovery period [16]. After LSG, patients who are severely obese have also shown a notable improvement in their quality of life [10,17]. LSG has been shown through a thorough review of the literature to be an effective and successful technique for improving reproductive function in women who suffer from extreme obesity [18]. Moreover, it has been shown that BS improves menstrual cyclicity in anovulatory cycle [19].

This study aims to examine how SG affects the menstrual cycle, with particular attention to hormonal dynamics and consequences for fertility.

## Materials and methods

Study design

A cross-sectional study was conducted among women in Saudi Arabia post-SG between December 2023 and May 2024, using an electronic online survey.

Study population

Women from all regions of Saudi Arabia who had undergone SG surgery participated in the study.

Sample size

The sample size was calculated based on the Raosoft Sample Size Calculator (http://www.raosoft.com/samplesize.html) [20] for sample size estimation, so by applying this formula, the estimated sample size was calculated as 385. With a margin of error of 5%, a confidence level of 95%, and a sample proportion of 50%, 385 individuals in the general population were selected.

Inclusion and exclusion criteria

Our study's inclusion criteria include any woman in Saudi Arabia aged between 18 and 55 years, as well as any woman who underwent SG surgery. On the other hand, the exclusion criteria consist of an uncompleted questionnaire, and any women have not accepted to participate.

Data collection

Data were gathered using a structured questionnaire created to assess women who underwent SG and have menstrual changes in Saudi Arabia. The study took into account many variables such as sociodemographic characteristics, prevalence rates of changes in the menstrual cycle after SG, and the association between SG and changes in the menstrual cycle.

The first part of the questionnaire covers the sociodemographic information of the study participants such as age, marital status, and region.

The second part about details of the SG operation involved asking about sleeve surgery history, and weight loss after the surgery.

The third part of the questionnaire is about the medical history and menstrual history mentioned in its chronic diseases, also if she used contraception. In menstrual history, we asked about any changes in the menstrual cycle and complications related to the menstrual cycle, such as pain or severe bleeding before the operation, and any changes noticed after the operation.

In the last part, we assessed the quality of life by using the World Health Organization Quality of Life: Brief Version (WHOQOL-BREF) score system. The WHOQOL-BREF is a 26-item instrument that measures quality of life across four domains: physical health, psychological health, social relationships, and environmental health. Each item is scored from 1 to 5, and the scores are linearly transformed to a 0-100 scale. The physical health domain covers aspects such as mobility, daily activities, and energy, while the psychological domain assesses self-image, positive attitudes, and mental status. The social relationships domain includes personal relationships and social support, and the environmental domain covers issues such as financial resources, living environment, and transportation [21].

Pilot study

We conducted the pilot study on 10% of the total sample of women undergoing SG and menstrual changes in Saudi Arabia to confirm the necessary adjustments and ensure the clarity of the tools. It served as a rough approximation of the time required to identify any issues with data collection.

Data management

The data were kept in Statistical Product and Service Solutions (SPSS, version 22; IBM SPSS Statistics for Windows, Armonk, NY) without identifying the participants. Thus, the questionnaire would not have any personal information on it, such as a name, an ID number, or anything else that could be used to find the subject.

Data analysis

We entered the data into Excel (Microsoft® Corp., Redmond, WA). Then, we transferred the data to the SPSS program. The sample was summarized using descriptive statistics, which included frequencies, percentages, means, and standard deviations. We analyzed the data by provider type when appropriate, and used the Pearson chi-square test to compare observed data. Differences between pretest and posttest were evaluated using the two-sample T-test. P values were considered significant if they were less than 0.05.

Ethical consideration

Patients' confidentiality and data privacy are priorities. Therefore, we did not use any information that could raise ethical concerns, such as participant names. Ethical approval was given by King Khalid University's medical college's ethical committee (Ecm#2023-2043).

## Results

Our study included 803 participants, of which 387 were included and the remaining were excluded based on exclusion criteria (Table 1). A significant proportion of participants were aged between 26 and 35 years (n=147, 38.0%), and those aged 36-45 years constituted 30.7% (n=119). The majority of participants were married (n=230, 59.4%), while 40.6% were single (n=157). Regional distribution showed that most participants were from the south (n=139, 35.9%), followed by the central region (n=74, 19.1%), east (n=70, 18.1%), west (n=66, 17.1%), and north (n=38, 9.8%). Additionally, 69.8% (n=270) reported not suffering from any chronic disease, whereas 30.2% (n=117) reported having chronic conditions.

**Table 1:** Sociodemographic and other parameters of participants

Variable		Frequency (n=387)	Percent
Age	18-25 Years	72	18.6
	26-35 Years	147	38.0
	36-45 Years	119	30.7
	46-55 Years	49	12.7
Marital Status	Single	157	40.6
	Married	230	59.4
Region	South	139	35.9
	Central	74	19.1
	East	70	18.1
	West	66	17.1
	North	38	9.8
Do You Suffer From Any Chronic Diseases?	No	270	69.8
	Yes	117	30.2

**Figure 1:**
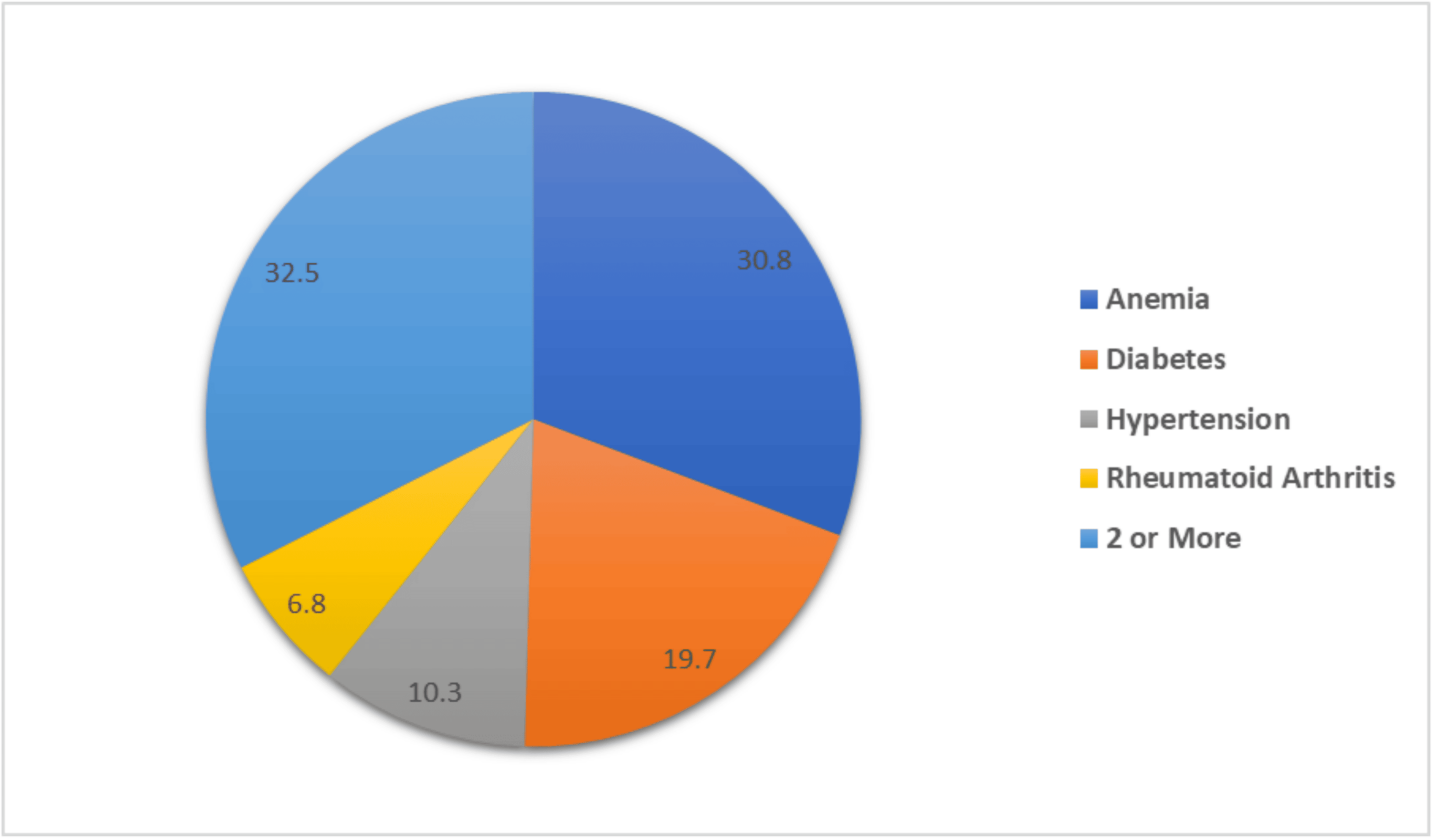
Different comorbidities among participants (N=117)

Figure 1 shows the different comorbidities among participants (n=117). Notably, the distribution of different conditions shows that anemia was the most common comorbidity, affecting 30.8% (n=36) of the participants. Diabetes was present in 19.7% (n=23), while hypertension affected 10.3% (n=12). Rheumatoid arthritis was reported by 6.8% (n=8) of the participants. Additionally, a significant portion, 32.5% (n=38), had two or more comorbid conditions.

Table 2 shows the prevalence of menstrual changes and related features in women post-SG. Most participants had the surgery over a year ago (n=227, 58.7%), with fewer having it six months to one year (n=68, 17.6%), three-six months (n=53, 13.7%), or less than three months prior (n=39, 10.1%). A significant majority experienced notable weight loss post surgery (n=336, 86.8%). Contraceptive use was low, with only 16.0% (n=62) used contraceptives. Before surgery, 62.5% (n=242) had regular menstrual cycles, whereas 37.5% (n=145) had irregular cycles. About half had experienced menstrual changes prior to surgery (n=195, 50.4%), and 31.3% (n=121) reported menstrual problems. Post surgery, 70.5% (n=273) noticed changes in their menstrual cycle, while 29.5% (n=114) did not.

**Table 2:** Prevalence of menstrual changes after sleeve gastrectomy and other features

Variable		Frequency (n=387)	Percent
How long have you had gastric sleeve surgery?	<3 Months	39	10.1
	3-6 Months	53	13.7
	6 Months-1 Year	68	17.6
	>1 Year	227	58.7
Did you experience significant weight loss after surgery?	No	51	13.2
	Yes	336	86.8
Do you use any contraceptives?	No	325	84.0
	Yes	62	16.0
Before gastric sleeve surgery, how do you describe your menstrual cycle?	Irregular	145	37.5
	Regular	242	62.5
Have you experienced changes in your menstrual cycle before?	No	192	49.6
	Yes	195	50.4
Did you have any menstrual problems before surgery?	No	266	68.7
	Yes	121	31.3
Have you noticed any changes in your menstrual cycle since gastric sleeve surgery?	No	114	29.5
	Yes	273	70.5

**Figure 2:**
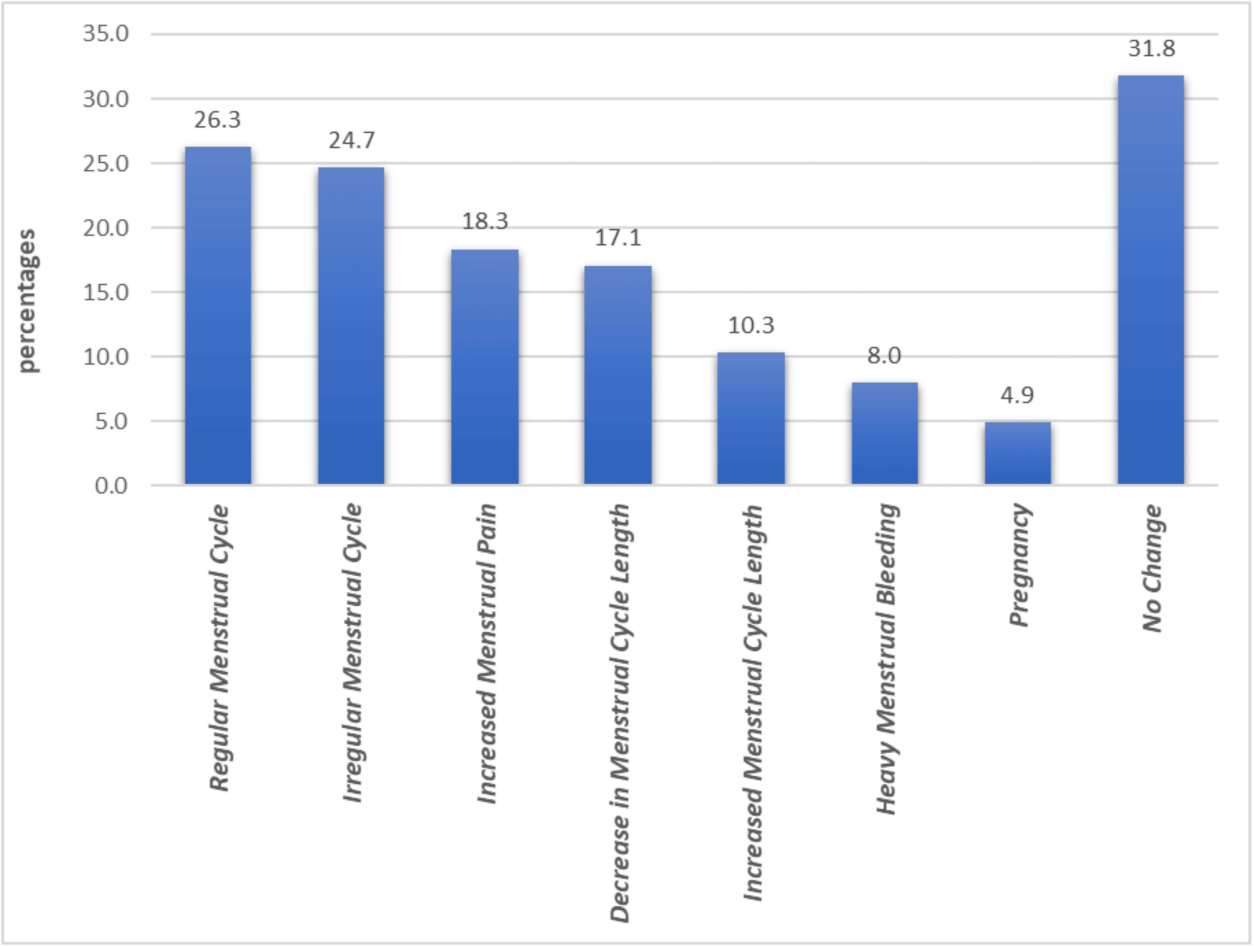
Different impacts of sleeve gastrectomy on menstrual regularity, cycle length, and associated symptoms

Figure 2 shows the impacts of sleeve gastrectomy on menstrual regularity, cycle length, and associated symptoms. A significant proportion of women reported experiencing a regular menstrual cycle post surgery (n=102, 26.3%), followed by irregularity in the menstrual cycle (n=96, 24.7%), while increased menstrual pain was reported by 18.3% (n=71) of participants. Additionally, 17.1% (n=66) noticed a decrease in menstrual cycle length. On the other hand, 10.3% (n=40) observed an increase in cycle length. Heavy menstrual bleeding affected 8% (n=31) of the women, and 4.9% (n=19) reported pregnancy. Notably, 31.8% (n=123) did not notice any changes in their menstrual cycle following the surgery.

Table 3 shows the assessment of patients over the past two weeks. Physical pain did not prevent 26.9% (n=104) from doing things that they wanted to do, while 4.1% (n=16) felt extreme hindrance. Enjoyment of life was high, with 37.5% (n=145) enjoying life a lot and 18.1% (n=70) extremely so. Concentration was moderate for 36.7% (n=142), and 27.4% (n=106) felt able to concentrate a lot. Regarding their environment, 36.7% (n=142) considered it moderately healthy, and 19.1% (n=74) considered it very healthy. Energy levels for daily life were moderate in 44.7% (n=173), and 23.5% (n=91) had a lot of energy. Information availability was moderate for 35.7% (n=138) and high for 34.9% (n=135). Engagement in recreational activities was moderate for 33.6% (n=130) and a lot for 22.2% (n=86). Movement ease was high for 39.0% (n=151), with 16.8% (n=65) finding it extremely easy.

**Table 3:** Assessment of patient about the extent to do or exposure to certain things over the past two weeks

Variable		Response				
		Nothing	A Little	Moderate	Alot	Extreme
To what extent do you feel that physical pain prevents you from doing the things you want to do?	N	104	109	111	47	16
	%	26.9	28.2	28.7	12.1	4.1
To what extent do you enjoy life?	N	15	41	116	145	70
	%	3.9	10.6	30.0	37.5	18.1
To what extent are you able to concentrate?	N	13	88	142	106	38
	%	3.4	22.7	36.7	27.4	9.8
To what extent do you consider your surrounding environment healthy?	N	38	103	142	74	30
	%	9.8	26.6	36.7	19.1	7.8
Do you have enough energy to go about daily life?	N	16	72	173	91	35
	%	4.1	18.6	44.7	23.5	9.0
How available is the information you need in your daily life?	N	15	54	138	135	45
	%	3.9	14.0	35.7	34.9	11.6
To what extent do you have the opportunity to engage in recreational activities?	N	28	107	130	86	36
	%	7.2	27.6	33.6	22.2	9.3
How easily are you able to move around?	N	22	55	94	151	65
	%	5.7	14.2	24.3	39.0	16.8

**Figure 3:**
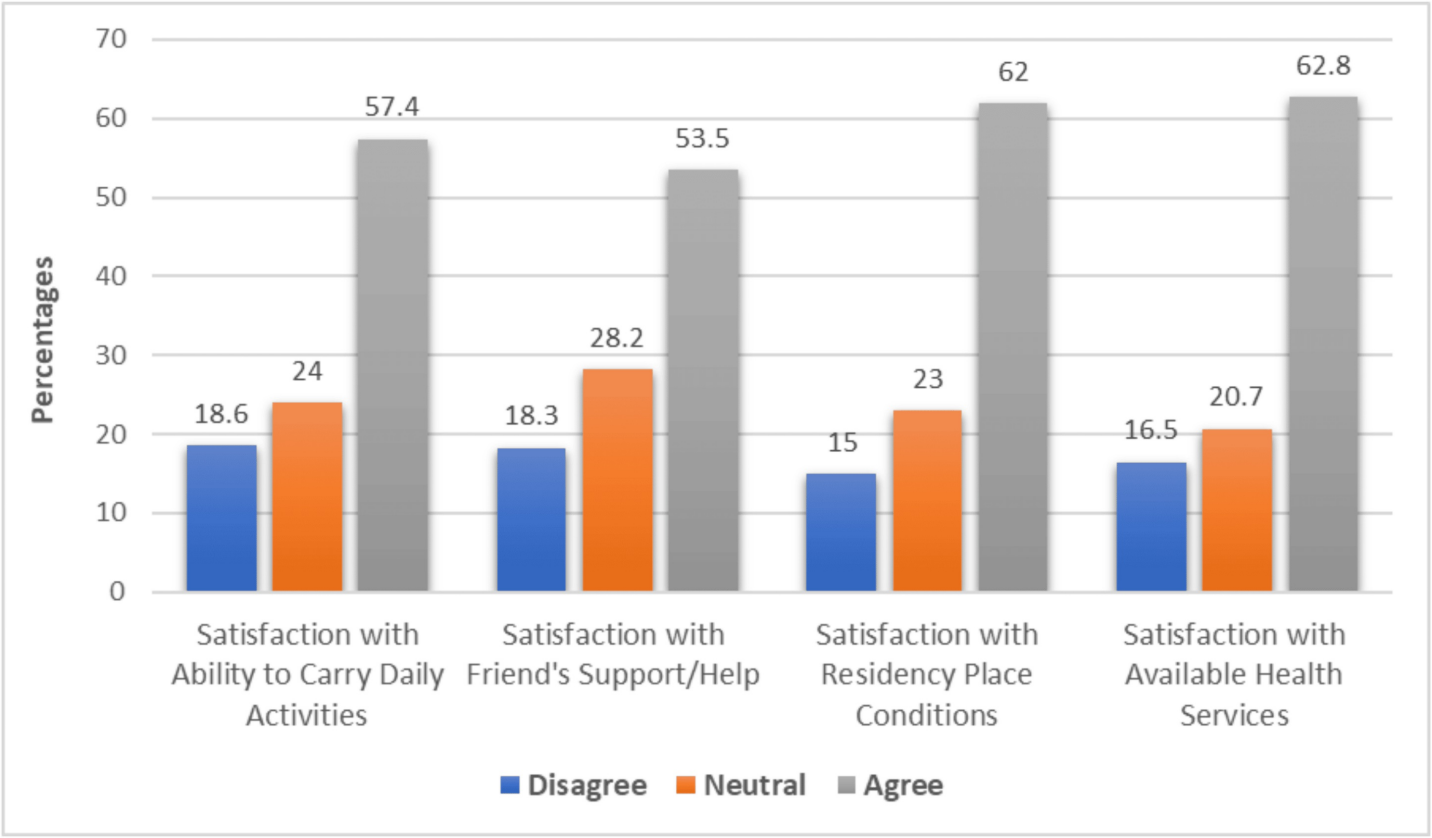
Different impacts of sleeve gastrectomy on menstrual regularity, cycle length, and associated symptoms

Figure 3 shows the impacts of sleeve gastrectomy on various aspects related to satisfaction among participants. For satisfaction with the ability to carry out daily activities, 18.6% (n=72) disagreed, 24% (n=93) were neutral, and 57.4% (n=222) agreed. Regarding satisfaction with friends' support or help, 18.3% (n=71) disagreed, 28.2% (n=109) were neutral, and 53.5% (n=207) agreed. Satisfaction with residency place conditions showed that 15% (n=58) disagreed, 23% (n=89) were neutral, and 62% (n=240) agreed. Lastly, satisfaction with available health services indicated that 16.5% (n=64) disagreed, 20.7% (n=80) were neutral, and 62.8% (n=243) agreed.

Table 4 shows the result of binary logistic regression analysis, which identified several predictors for experiencing menstrual changes after sleeve gastrectomy. Age showed a significant negative association (B = -0.321, p-value = 0.028), indicating that younger age groups are less likely to experience menstrual changes (Exp(B) = 0.725, 95% CI: 0.545-0.965). Marital status (B = 0.272, p-value = 0.329) and time since surgery (B = 0.025, p-value = 0.841) were not significant predictors. Having a chronic disease (B = 0.318, p-value = 0.242), using contraceptives (B = -0.183, p-value = 0.583), significant weight loss (B = 0.392, p-value = 0.249), and a regular menstrual cycle before surgery (B = -0.416, p-value = 0.195) were not significant predictors. However, having changes in the menstrual cycle before surgery was a significant predictor (B = 0.926, p-value = 0.002), increasing the likelihood of post-surgery changes (Exp(B) = 2.525, 95% CI: 1.394-4.575). Having menstrual problems before surgery (B = -0.258, p-value = 0.376) was not significant.

**Table 4:** Different adjusted predictors of experiencing menstrual changes after surgery (binary logistic regression) (*) Significant value

Variable	B	P-value	Exp(B)	95% CI	
				Lower	Upper
Age	-0.321	0.028*	0.725	0.545	0.965
Marital Status (Married)	0.272	0.329	1.313	0.760	2.267
Time Since Gastric Sleeve Surgery	0.025	0.841	1.025	0.806	1.304
Suffering from any Chronic Disease	0.318	0.242	1.375	0.806	2.343
Use Any Contraceptives	-0.183	0.583	0.833	0.434	1.599
Significant Weight Loss After Surgery	0.392	0.249	1.480	0.760	2.883
Regular Mensural Cycle Before Surgery	-0.416	0.195	0.659	0.351	1.238
Changes in Menstrual Cycle Before Surgery	0.926	0.002*	2.525	1.394	4.575
Any Menstrual Problem Before Surgery	-0.258	0.376	0.773	0.437	1.368

Table 5 shows the association between regions and changes in the menstrual cycle after sleeve gastrectomy. In the south, 71.9% (n=100) experienced changes, while 28.1% (n=39) did not. In the central region, 59.5% (n=44) experienced changes, and 40.5% (n=30) did not. In the east, 72.9% (n=51) had changes, with 27.1% (n=19) reporting no changes. The west showed the highest percentage of changes, with 78.8% (n=52) experiencing changes and 21.2% (n=14) not experiencing changes. In the north, 68.4% (n=26) reported changes, and 31.6% (n=12) did not. The significance value for the association was 0.140, indicating no statistically significant association between region and menstrual cycle changes post surgery.

**Table 5:** Association between regions and changes after surgery ^a^Chi-square test

Variable		Any Changes in Your Menstrual Cycle Since Gastric Sleeve Surgery?		P-value
		No	Yes	
South	N	39	100	0.140^a^
	%	28.1%	71.9%	
Central	N	30	44	
	%	40.5%	59.5%	
East	N	19	51	
	%	27.1%	72.9%	
West	N	14	52	
	%	21.2%	78.8%	
North	N	12	26	
	%	31.6%	68.4%	

**Figure 4:**
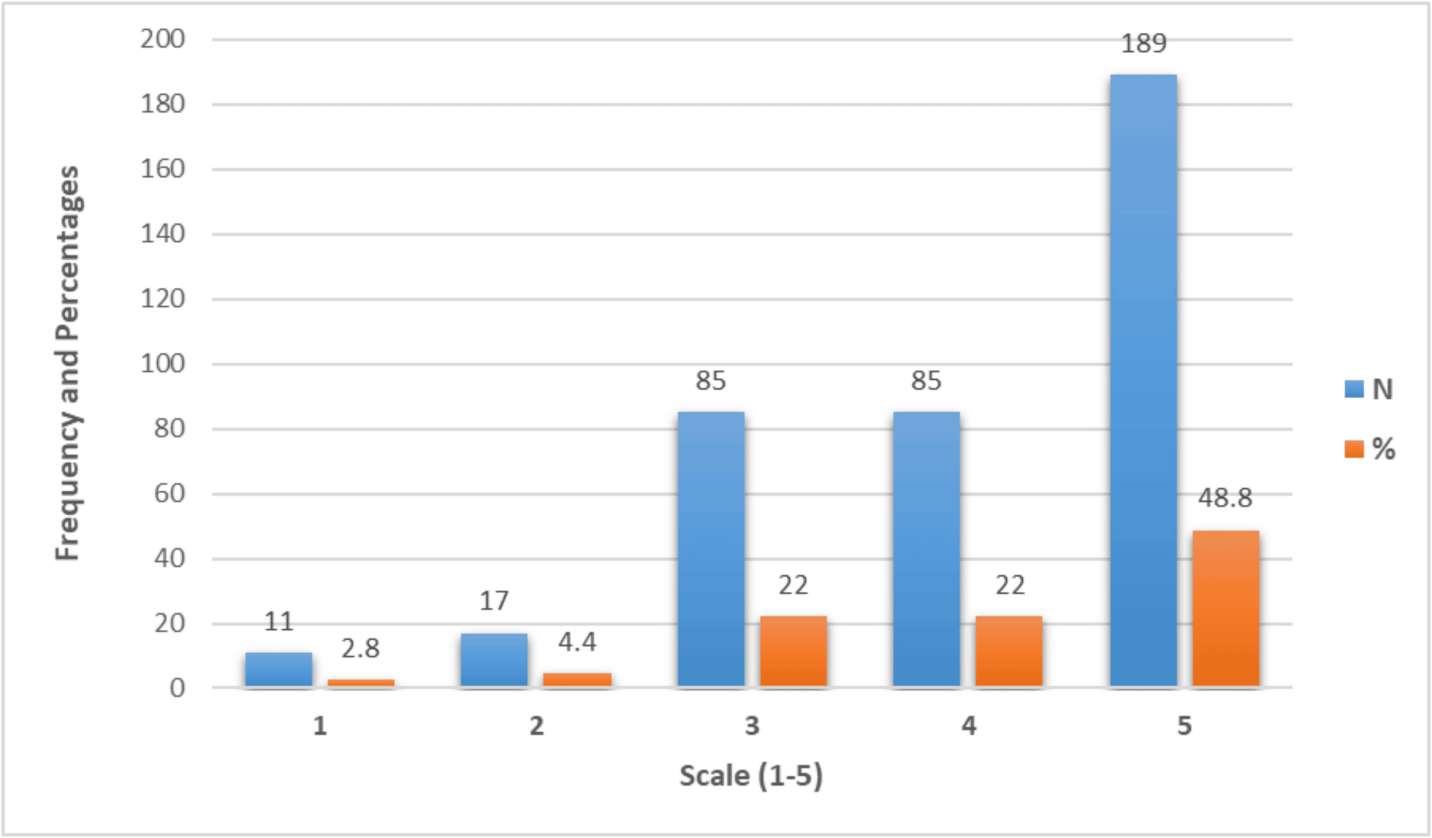
Quality of life according to patients’ perception after surgery (scale 1-5)

Figure 4 depicts the quality of life according to patients’ perception after sleeve gastrectomy, measured on a scale from 1 to 5. The majority of patients rated their quality of life highly, with 48.8% (n=189) giving it a 5 and 22% (n=85) giving it a 4. A moderate quality of life rating of 3 was also reported by 22% (n=85). Lower ratings were less common, with 4.4% (n=17) rating their quality of life as 2 and 2.8% (n=11) rating it as 1.

## Discussion

Worldwide, there are about 650 million obese persons, and since 1975, the number has increased, especially among women and older elderly. In a similar vein, Alsulami et al. reported that, in 2016, over 1.9 billion adult individuals (or 39% of the total population) were overweight, and over 650 million (or 13%) were obese, including 10.8% of men and 14.9% of women [22]. Complex connections between genetics, environment, and lifestyle are involved. Common-form obesity is supported by both hereditary and environmental variables, according to Qi et al. [23]. BS corrects menstruation abnormalities and hormonal imbalances to enhance quality of life and reproductive health. Furthermore, Wolfe et al. showed that BS likely improves cardiovascular and overall survival more than lifestyle changes due to substantial weight loss and potential neuroendocrine effects [24]. Moreover, Moxthe et al. showed that BS considerably improved hormonal balance and sexual functions in females and improved the chance of conception in females [25]. Our study aimed to explore the prevalence of menstrual changes amongst Saudi women after sleeve gastrectomy and associated factors that could influence quality of life and other health parameters.

Notably, our study revealed that a substantial proportion of women experienced menstrual changes after sleeve gastrectomy. Specifically, 70.5% of participants reported changes in their menstrual cycle post surgery. However, previous studies show that there is a positive impact of surgery on reproductive health and the menstrual cycle due to weight loss and metabolic changes. Similarly, Różańska-Walędziak et al. showed that before the surgery, 38.6% of the patients reported irregular menstruations in comparison with 25.0% after BS [11]. Moreover, Alhumaidan et al. showed that, after surgery, menstrual irregularities were reduced from 41.9% to 36.2%, indicating a positive effect of BS [8]. Notably, 26.3% observed menstrual cycle regularity, and 4.9% become pregnant after surgery. Similarly, Teitelman et al. showed that menstrual cycle disorders may completely resolve after BS [12]. These findings align with the known physiological responses to significant weight loss and metabolic shifts induced by BS.

In our study, binary logistic regression analysis identified several predictors for experiencing menstrual changes post surgery. Age was found to be a significant predictor, with younger women being less likely to experience menstrual changes. This observation contrasts with previous literature suggesting that older age might correlate with greater hormonal disruptions post BS. However, a study by Cai et al. showed that there is no impact of age on the menstrual outcome after surgery [26]. Interestingly, having experienced changes in menstrual cycles before surgery significantly predicted post-operative changes, emphasizing the role of pre-existing hormonal profiles in surgical outcomes.

Notably, the prevalence of comorbidities was high among participants, with anemia being the most common (30.8%), followed by diabetes (19.7%) and hypertension (10.3%). These comorbidities are known to impact both surgical outcomes and menstrual health, potentially complicating the metabolic adjustments following sleeve gastrectomy. Similarly, Fowler et al. showed that chronic conditions significantly increase surgical risks, leading to higher mortality and readmission rates, necessitating tailored strategies such as enhanced recovery pathways for improved perioperative care [27]. Future research should explore how managing these conditions pre- and post-operatively influences menstrual health outcomes.

Assessment of quality of life post surgery revealed generally positive perceptions among participants. A majority of participants reported high satisfaction with their ability to carry out daily activities, as well as with social support and health services post-surgery. The perception of improved quality of life following sleeve gastrectomy is consistent with existing literature highlighting the significant weight loss and health improvements experienced by many patients. Similarly, Sierżantowicz et al. showed that bariatric treatment seems to provide a persistent benefit in terms of Health-Related Quality of Life (HRQOL), especially its physical component score [28].

Chronic conditions significantly impact surgical outcomes, as it is a well-established fact that the presence of comorbidity always compromises surgical outcomes. For example, coexisting medical conditions such as anemia, diabetes, and hypertension can have a substantial effect on the results of surgical procedures and the overall well-being of the menstrual cycle. Anemia can result in heightened surgical complications and disruptions in menstrual patterns. Diabetes can impede the process of wound healing and elevate the likelihood of infection, whereas hypertension can complicate the administration of anesthesia and the subsequent recovery, potentially increasing the risks of complications and affecting recovery. Tailored perioperative strategies are essential to manage these complexities and improve patient outcomes, despite challenges in defining high-risk profiles based solely on disease count. Enhanced recovery pathways and targeted interventions may mitigate risks and enhance the efficacy of surgical interventions for patients with multi-morbidity.

Limitations and future directions

Considering the insightful discoveries made, our study has a number of limitations. Firstly, our ability to determine the causal relationships between surgical factors and menstrual changes is limited by the cross-sectional design. A deeper understanding of how menstrual health changes over time after surgery would be possible with longitudinal research. Secondly, the utilization of self-reported data may lead to biases such as subjective interpretations of quality of life and menstrual changes, as well as recall bias.

Implications and future direction

Our study emphasizes the necessity of tailored pre-operative counseling for women undergoing sleeve gastrectomy, especially those with existing menstrual irregularities. Healthcare providers should prioritize monitoring and support to effectively manage post-operative outcomes. Future research should investigate the hormonal and metabolic mechanisms underlying these changes, conduct longitudinal studies to assess durability and impact on reproductive health, and compare outcomes across diverse populations to enhance clinical guidelines and patient care in BS settings.

## Conclusions

Our research adds to the expanding corpus of literature on the impact of sleeve gastrectomy on menstrual health and quality of life among Saudi women. The findings underscore the need for comprehensive pre-operative counseling, personalized management strategies, and ongoing support to address menstrual changes and optimize overall health outcomes post surgery. By understanding these dynamics, healthcare providers can better tailor interventions to meet the unique needs of BS patients, improving their general state of health and living standards.
